# Impact of Chronic Heart Failure on Vascular Responsiveness: A Prospective Observational In Vivo and Ex Vivo Study in Patients Undergoing Cardiac Surgery †

**DOI:** 10.3390/jcm15208179

**Published:** 2026-10-21

**Authors:** Olga Papazisi, Marieke E. van Vessem, Saskia L. M. A. Beeres, Rob B. P. de Wilde, Remco R. Berendsen, René de Vries, Bart J. A. Mertens, Carlos Elzo Kraemer, Jan H. N. Lindeman, Robert J. M. Klautz, Martin C. Michel, A. H. Jan Danser, Meindert Palmen

**Affiliations:** 1 Department of Cardiothoracic Surgery, Leiden University Medical Center, 2300 RC Leiden, The Netherlands; 2 Department of Cardiothoracic Surgery, University Medical Center Utrecht, 3508 GA Utrecht, The Netherlands; 3 Department of Sports Medicine, Maxima Medical Center, 5500 MB Veldhoven, The Netherlands; 4 Department of Cardiology, Leiden University Medical Center, 2300 RC Leiden, The Netherlands; 5 Department of Intensive Care, Leiden University Medical Center, 2300 RC Leiden, The Netherlands; rdwilde42@gmail.com (R.B.P.d.W.);; 6 Department of Anesthesiology, Leiden University Medical Center, 2300 RC Leiden, The Netherlands; 7 Division of Pharmacology and Vascular Medicine, Department of Internal Medicine, Erasmus MC University Medical Center, 3000 CA Rotterdam, The Netherlands; 8 Department of Biomedical Data Sciences, Leiden University Medical Center, 2300 RC Leiden, The Netherlands; 9 Department of Vascular Surgery, Leiden University Medical Center, 2300 RC Leiden, The Netherlands; 10 Department of Pharmacology, University Medical Center, Johannes Gutenberg University, 55131 Mainz, Germany

**Keywords:** heart failure, vasoplegia, vasoconstriction, cardiac surgery, α_1_-adrenergic receptors

## Abstract

**Background**: Patients with chronic heart failure (HF) are at increased risk of vasoplegia after cardiac surgery, potentially because neurohormonal activation in the context of HF alters vascular regulation. **Methods**: We compared perioperative vasoresponsiveness in patients with and without HF undergoing cardiac surgery with cardiopulmonary bypass (CPB). Patients were prospectively enrolled, and changes in systemic vascular resistance (ΔSVR) after phenylephrine and nitroglycerin were measured perioperatively (in vivo). Ex vivo vasoreactivity to phenylephrine and vasopressin was tested in 40 biopsies. **Results**: In vivo and ex vivo analyses included 36 and 13 patients (10 controls and 3 HF patients), respectively. At baseline, phenylephrine increased SVR by 26% in HF patients (estimated marginal mean, 95% CI 16–35%) versus 51% in controls (95% CI 38–64%; *p* = 0.002). This attenuated response persisted during CPB, and ex vivo observations suggested a similar pattern. Responsiveness to nitroglycerin was unchanged. Lower post-CPB ΔSVR was associated with greater norepinephrine use during the first postoperative day (β = −346.6, 95% CI −541.2 to −151.9; R^2^ = 0.291). Conversely, HF patients showed greater ex vivo responsiveness to vasopressin. **Conclusions**: HF is associated with marked impairment of α_1_-adrenergic vasoresponsiveness, indicating impaired α_1_-adrenergic signaling. Preserved vasopressin responsiveness may support its earlier use for postoperative hypotension in these patients.

## 1. Introduction

When optimal medical therapy fails to prevent progression of heart failure (HF), cardiac interventions and HF surgery may be required [1]. However, the use of cardiopulmonary bypass (CPB) and induction of surgical trauma increase vasoplegia susceptibility in this vulnerable patient population, with incidence rates up to 66% [2]. Vasoplegia is characterized by systemic hypotension and high vasopressor requirements despite normal or high cardiac output (CO) and is associated with increased morbidity and mortality [3,4].

The etiology of vasoplegia pathophysiology is likely multifactorial in nature, with systemic inflammation playing a central role, resulting in dysregulated vascular tone and potential resistance to vasopressors [5]. Downregulation or desensitization of α_1_-adrenergic receptors in resistance arterioles and venules may impair vascular smooth muscle contraction [6]. Chronic adrenergic overstimulation in HF, in the setting of reduced CO, may downregulate/desensitize α_1_-receptors, analogous to myocardial β-adrenergic receptor downregulation in HF patients [7,8,9]. Moreover, chronic vasopressin activation may deplete its endogenous stores, causing relative deficiency in HF [10], predisposing these patients to postoperative systemic hypotension and vasoplegia [11].

We hypothesize that altered vasoresponsiveness in HF, exacerbated by CPB-induced inflammation, impairs the response to exogenous vasopressors, increasing susceptibility to postoperative vasoplegia and associated comorbidities [10,12]. A better mechanistic understanding could aid in the development of preventive and therapeutic strategies. Therefore, we investigated (in vivo and ex vivo) vascular responsiveness in HF patients undergoing cardiac surgery compared to non-HF control patients.

## 2. Patients and Methods

### 2.1. Study Design

The “Vasoresponsiveness in patients with heart failure (VASOR)” study is a prospective observational study conducted at Leiden University Medical Center [10]. Vascular reactivity in patients with and without HF was compared in vivo and ex vivo (Figure S1). For the ex vivo protocol, pericardial fat containing resistance arterioles was collected intraoperatively; arterioles were isolated and analyzed at the Erasmus Medical Center (Rotterdam).

VASOR adhered to the Declaration of Helsinki. This study is registered in the Netherlands Trial Register (NTR5647). The institutional ethics committee approved the protocol and amendments (P14.298). Reporting followed STROBE [13].

### 2.2. Study Population

Adults scheduled for elective or urgent cardiac surgery through median sternotomy with CPB were screened. Patients undergoing cardiac surgery with comparable expected CPB and aortic cross-clamp duration were included and assigned to either the HF or control group. Inclusion for HF: (1) chronic HF diagnosis [14]; (2) LVEF ≤ 35%. Inclusion for controls: (1) no HF; (2) LVEF > 50%. Exclusion criteria: (1) age < 18 years; (2) incapacitated adults; (3) emergency surgery; (4) preoperative need for moderate/high intravenous inotropic support (>4 µg/kg/min dobutamine or dopamine), preoperative vasopressor and/or mechanical support; (5) aortic regurgitation > grade 1 due to potential effect on intraoperative hemodynamic measurements (not for ex vivo); (6) preoperative use of nitrates, α_1_-blockers, or PDE-5 inhibitors; (7) endocarditis/active infection; (8) acute HF. All patients provided written informed consent.

Initially, 36 patients in total were planned for both in vivo and ex vivo analyses. Due to challenges with tissue sampling and methodology, 24 additional patients were enrolled for the ex vivo experiments. Due to logistics, tissue was collected in 40 patients. Identical anesthesia and hemodynamic monitoring protocols were used in both groups (Supplementary Materials).

### 2.3. Surgical Procedures

Indication and timing for surgery were determined by the multidisciplinary Heart Team. HF procedures included mitral repair (mainly restrictive annuloplasty for functional mitral regurgitation; *N* = 16), surgical left ventricular restoration (Dor’s technique; *N* = 7), left ventricular assist device (LVAD) implantation (HeartWare Inc., Framingham, MA, USA; *N* = 6), or aortic/aortic valve surgery (*N* = 11). Concomitant tricuspid repair and coronary revascularization were performed as required. A heterogeneous HF group was intentionally included to assess HF-related alterations in vasoresponsiveness. Controls mainly underwent mitral valve repair for primary mitral regurgitation with or without concomitant procedures. Operations were performed with (or without for LVAD implantation) intermittent antegrade warm blood cardioplegia. On the day of surgery, no angiotensin-converting enzyme inhibitors, angiotensin receptor blockers, or diuretics were administered.

### 2.4. Study Outcomes

The primary endpoint was systemic vascular resistance (SVR) change (ΔSVR) after a standardized phenylephrine dose at baseline. SVR response to phenylephrine was used to characterize systemic vasoresponsiveness, rather than as a stand-alone criterion for determining the cause of postoperative hypotension. Secondary endpoints were: (1) ΔSVR after phenylephrine administration at other perioperative time points; (2) nitroglycerin dose required for 10% decrease in mean arterial pressure (MAP) both pre- and postoperatively; (3) correlation between ΔSVR after phenylephrine (pre-induction and intraoperatively) and postoperative norepinephrine use; (4) occurrence of vasoplegia (continuous vasopressor need—norepinephrine ≥ 0.2 µg/kg/min or terlipressin—with cardiac index ≥ 2.2 L/min/m^2^ for ≥12 h within the first 3 postoperative days); (5) ex vivo vasoconstriction (% of preconstriction) to phenylephrine and vasopressin.

### 2.5. Vascular Reactivity Test

#### 2.5.1. In Vivo

For vasoconstriction, an intravenous bolus of phenylephrine (selective α_1_-agonist) 2 μg/kg was administered via the central venous line, and the effect on hemodynamics was recorded at four time points: (T1) baseline; (T2) after anesthesia induction; (T3) 15 min after CPB cessation; (T4) postoperative day 1. This dose has previously been used for this purpose in healthy volunteers [15].

For vasodilation, intravenous nitroglycerin (10 μg) was administered peripherally; MAP and SVR effects were recorded. Doses increased to 20, 40, and 60 μg until a ≥10% MAP decrease was achieved. Vasodilation tests were performed at baseline and postoperative day 1.

Tests were performed only when the clinical condition allowed for an increase or decrease in MAP. Additional perioperative measurement details are provided in the Supplementary Materials.

#### 2.5.2. Ex Vivo

Prepericardial fat from the pleuropericardial area was collected at the start of surgery (before CPB initiation), preserved in Krebs solution, and transported cooled (≈4 °C) to Erasmus MC. Resistance arterioles were collected and analyzed [16]. The detailed ex vivo protocol is provided in the Supplementary Materials.

### 2.6. Statistical Analysis

The study was powered (90%) to detect a 400 dyn·s/cm^5^ difference in mean ΔSVR, assuming common SD of 350 dyn·s/cm^5^, using a two-sided Student’s *t*-test at α = 0.05 [17,18].

Continuous variables are presented as mean ± SD or median (interquartile range or range). Between-group differences were analyzed using unpaired Student’s *t*-tests for normal data and median tests otherwise. Due to small numbers, between-group testing for ex vivo outcomes was not performed. Categorical variables are presented as counts/percentages; differences were analyzed using the Chi-square or Fisher’s exact test. Pairwise deletion addressed missing data.

Generalized estimating equations with an independent working correlation structure assessed HF effects on in vivo vasoreactivity accounting for the repeating measurements. For the analysis, phenylephrine-induced changes in SVR were expressed as percentages relative to the absolute value before the administration of phenylephrine and were calculated as [(SVR after phenylephrine − SVR before phenylephrine)/SVR before phenylephrine] × 100. Percentage changes were used to account for differences in baseline vascular resistance between patients and facilitate the interpretation of proportional vasopressor responsiveness. Considering that the sample size calculation for this study was initially based on absolute values, a sensitivity analysis was performed including absolute changes in SVR (dyn·s·cm^−5^) to assess consistency across findings. This additional analysis utilized the same generalized estimating equations model structure. Patients with incomplete repeated measurements contributed observations at time points for which the outcome and all required model variables were available. The analysis was not repeated excluding patients who received norepinephrine to rule out possible confounding since the majority of the HF patients were receiving this medication. Results are presented as estimated marginal means with 95% confidence intervals (CI). No other adjusted analyses were performed due to the small sample size.

Univariable linear regression assessed whether vasoreactivity correlated to norepinephrine use in the first postoperative day; multivariable regression adjusted for HF and norepinephrine dosage at the respective time point.

Significance was set at *p* < 0.05, and *p*-values other than for the primary endpoint are descriptive. Analyses were conducted using SPSS (version 25.0, IBM Corp., Armonk, NY, USA) and R (version 4.3.1; R Foundation for Statistical Computing, Vienna, Austria).

## 3. Results

### 3.1. Study Population

Of 3958 adult cardiac surgery patients screened between 2016 and 2022, 383 met the eligibility criteria. Initially, 36 patients were enrolled for combined in vivo and ex vivo assessment. Because the ex vivo vascular experiments were unsuccessful in a subset of these patients, an additional 24 patients were enrolled for ex vivo assessment only. Of the 60 enrolled patients, 2 were excluded intraoperatively because they no longer met the inclusion criteria. The final study population therefore comprised 58 patients, including 28 patients with HF and 30 controls. The in vivo analyses were performed in the initial 36 patients, whereas the ex vivo analyses were performed in 40 patients. Accordingly, the in vivo and ex vivo analyses included 36 and 40 patients, respectively (Figures S2 and S3).

### 3.2. In Vivo

HF and controls were similar in age (68 [62–71] vs. 64 [59–69] years) and sex (men: 67% vs. 89%). HF patients had higher BMI (27 ± 4 vs. 24 ± 3 kg/m^2^), higher EuroSCORE II (9.8% [6.6–15.5] vs. 1.5% [1.1–2.7], mainly driven by reduced LVEF), lower preoperative MAP (83.1 ± 14.7 vs. 95.3 ± 17.2 mmHg), and higher white blood cell counts (9 ± 3.4 × 10^9^/L vs. 6.7 ± 1.7 × 10^9^/L). B-blocker (89% vs. 33%) and diuretic (89% vs. 28%) use was more prevalent in HF (Table 1). Procedural, CPB, and aortic cross-clamp durations were comparable (Table 2). A similar postoperative rise in inflammatory parameters was observed (Table 3). Despite more inotrope/vasopressor use, HF patients had lower MAP during the first 24 postoperative hours (72 ± 8 vs. 77 ± 5 mmHg; *p* = 0.037) (Table 3). Postoperative vasoplegia occurred in 1 control (*p* = 1.0).

### 3.3. Phenylephrine Responsiveness Pre- and Intraoperative

**Figure 1:**
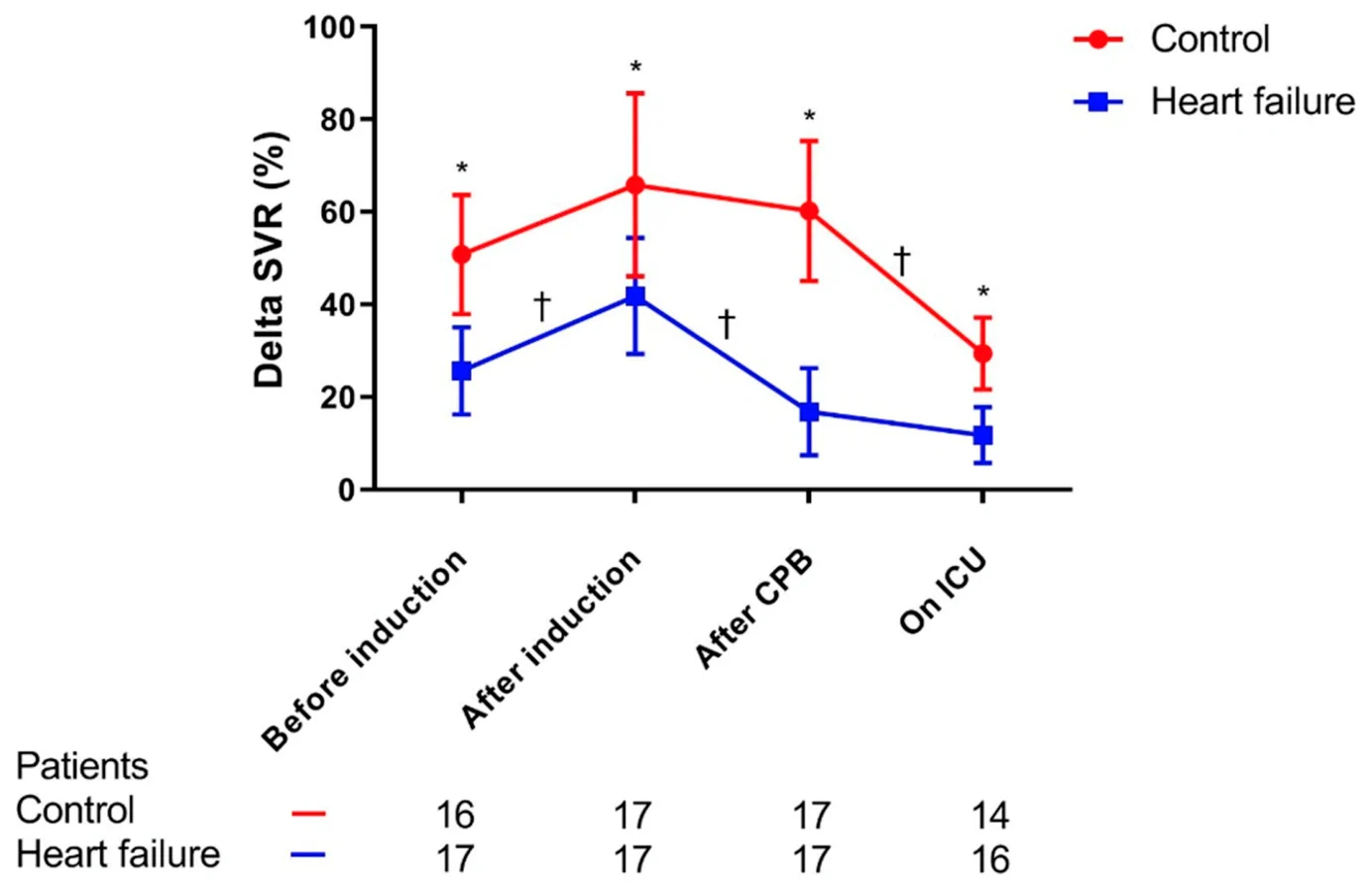
Vasoconstriction test in all patients. Delta systemic vascular resistance (ΔSVR) (%) with 95% CI. * *p* < 0.05 between groups; † between time points. T1: baseline, T2: after anesthesia induction, T3: after cessation of the cardiopulmonary bypass, T4: first postoperative day.

Phenylephrine-induced ΔSVR at baseline was lower in HF versus controls (Figure 1) [estimated mean 26% (95% CI 16; 35) vs. 51% (95% CI 38; 64), *p* = 0.002]. Sensitivity analysis using absolute changes in SVR yielded a between-group difference in the same direction as the primary percentage-based analysis (estimated difference 325.1 dyn·s·cm^−5^ (95% CI −554.7; −95.5), *p* = 0.006, Table S2).

After induction, both groups showed enhanced ΔSVR versus pre-induction (*p* = 0.013 HF; *p* = 0.058 controls), yet responses remained lower in HF (Figure 1). In controls, ΔSVR after CPB cessation remained similar to pre-CPB (estimated mean 66% [95% CI 46; 86] to 60% [95% CI 45; 75]; *p* = 0.623). In HF, vasoresponsiveness was nearly abolished post-CPB (estimated mean 42% [95% CI 29; 54] to 17% [95% CI 7; 26]; *p* = 0.002). Post-CPB ΔSVR correlated with higher norepinephrine requirements during the first postoperative day (β −346.6 [95% CI −541.2; −151.9], R^2^ = 0.291) (Table 4). Reduced phenylephrine responsiveness in HF was of cardiac index at each time point (Figure S4A). Moreover, the correlation with postoperative norepinephrine requirements remained after adjustment for HF and norepinephrine dose (β −270.2 [95% CI −519.3; −21.1], R^2^ = 0.361).

### 3.4. Early Postoperative Vasoresponsiveness

The diminished phenylephrine responsiveness observed in HF post-CPB (ΔSVR, estimated mean 17%) persisted postoperatively (ΔSVR, estimated mean 12% [95% CI 6; 18]; *p* = 0.026 vs. baseline). In controls, vasoresponsiveness also declined postoperatively (from estimated mean 60% intraoperatively to 29% [95% CI 22; 37%] in the ICU; *p* < 0.001 vs. baseline) but remained higher than in HF.

MAP responses paralleled SVR findings (Figure S3). In controls, phenylephrine consistently increased MAP intraoperatively (more after induction), with postoperative attenuation. In HF, MAP increases were blunted perioperatively and remained significantly lower in the ICU.

### 3.5. Vasoresponsiveness to Nitroglycerin

Despite between-group differences in phenylephrine responsiveness, nitroglycerin produced similar effects: both groups required comparable doses to achieve ≥10% MAP reduction at baseline and on postoperative day 1 (Figure S4B).

### 3.6. Ex Vivo

Preoperative characteristics are presented in Table S1. We processed 40 biopsies; 13 experiments were successful (10 controls, 3 HF, Figure S3). Exclusions were due to absent/minimal KCl response (*N* = 7), spontaneous contractility (*N* = 4), or absence of usable vessels (*N* = 16).

**Figure 2:**
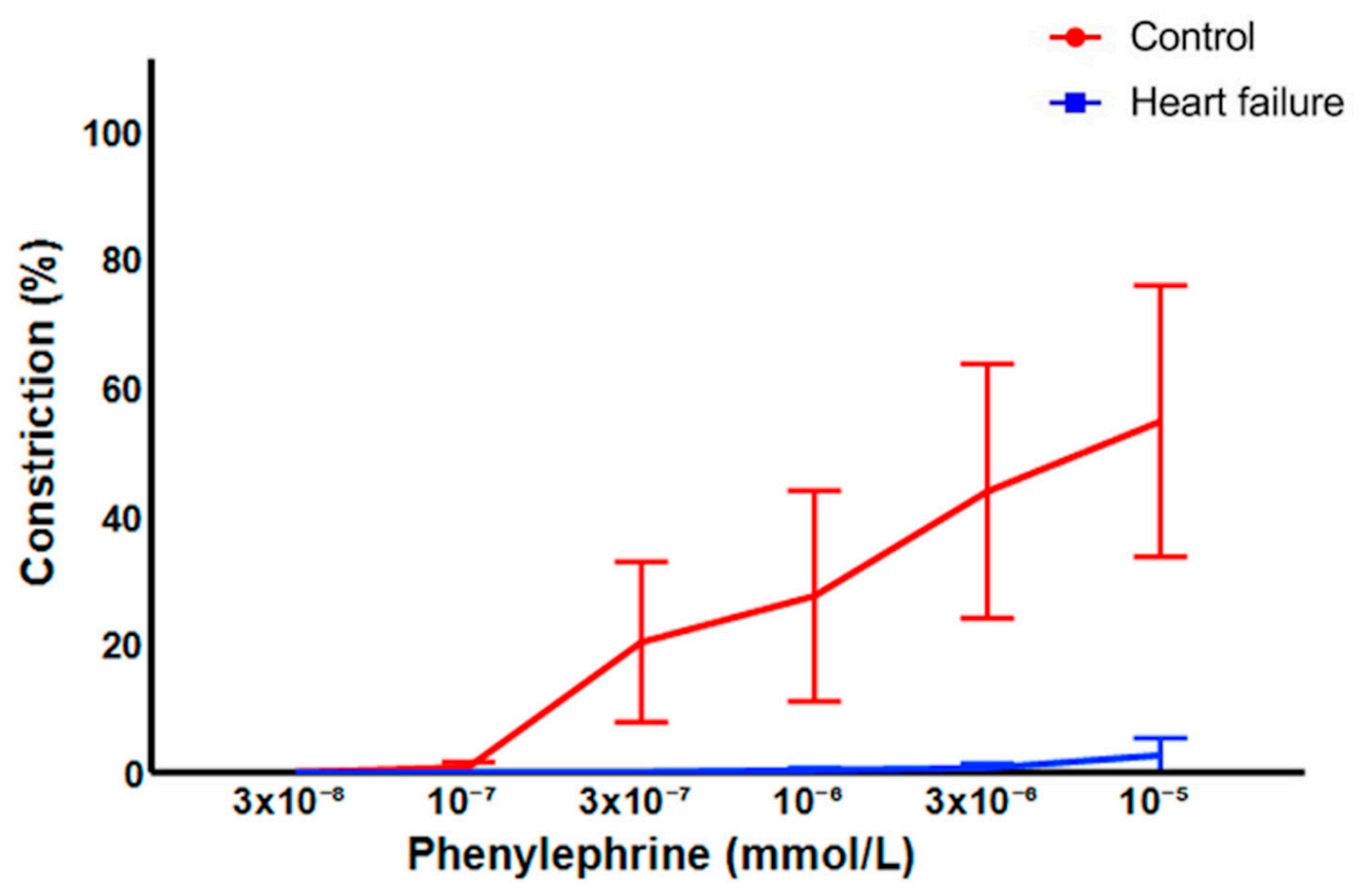
Vascular responsiveness to phenylephrine ex vivo. The figure represents the % of the 100 mM KCl-induced constrictions after incrementing dosages of phenylephrine in mmol/L. The lines depict the mean ± standard error. Heart failure, N: 3, control, N: 10.

**Figure 3:**
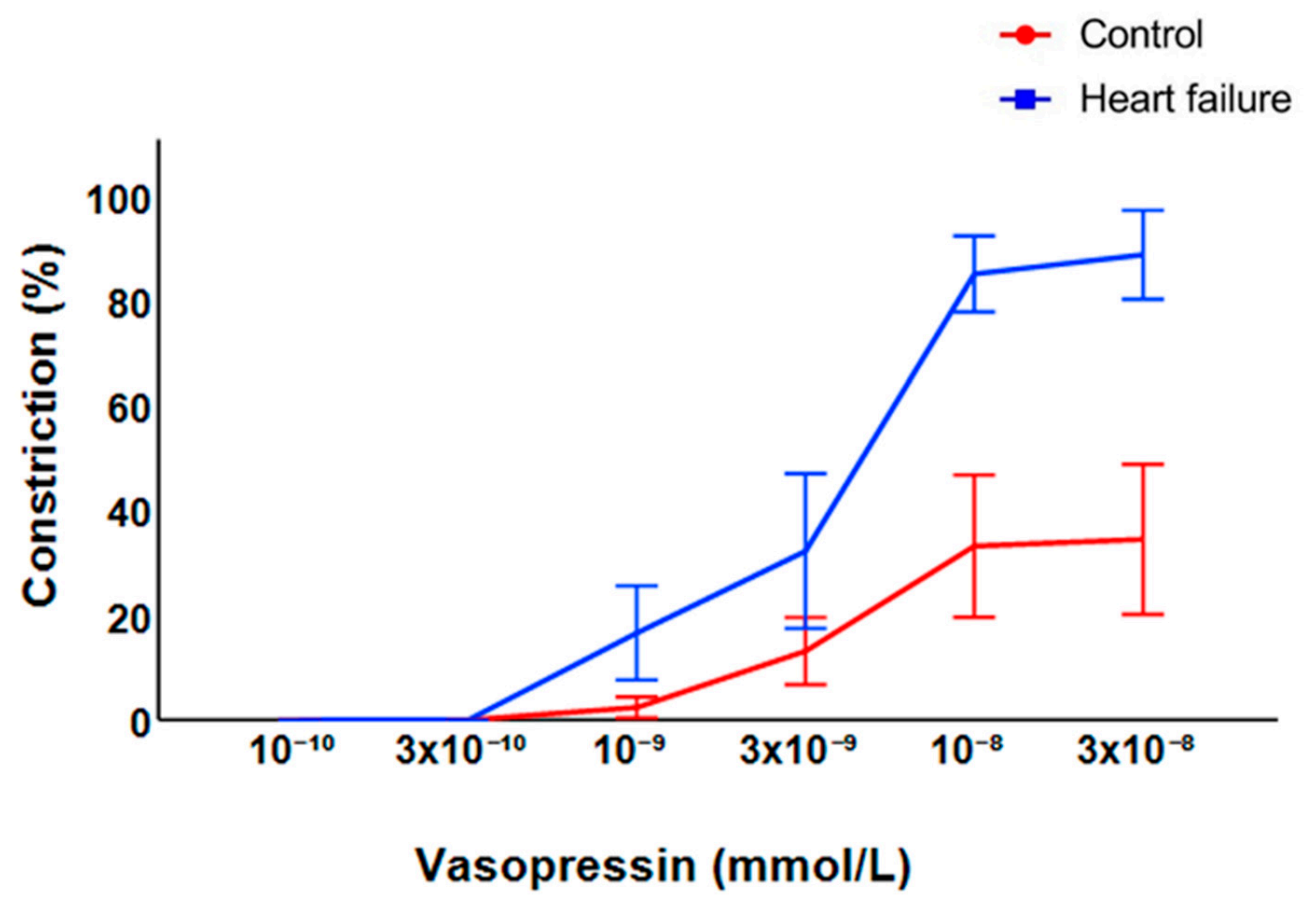
Vascular responsiveness to vasopressin ex vivo. The figure represents the % of the 100 mM KCl-induced constrictions after incrementing dosages of vasopressin in mmol/L. The lines depict the mean ± standard error. Heart failure, N: 3, control, N: 10.

HF arterioles exhibited abolished responses to phenylephrine (Figure 2), whereas controls showed concentration-dependent constriction. Conversely, HF arterioles demonstrated increased responsiveness to vasopressin compared with controls (Figure 3).

## 4. Discussion

HF patients undergoing cardiac surgery displayed a markedly diminished response to phenylephrine compared with controls during the in vivo and ex vivo protocols. In contrast, HF patients exhibited increased ex vivo vasopressin sensitivity. Considering that various vasoconstrictors eventually use the same cellular machinery to elicit vascular smooth muscle cell contraction, the directionally opposite responses seen in our study—reduced phenylephrine yet increased vasopressin responsiveness—argue against a nonspecific impairment of contraction and point instead towards a specific defect in α_1_-receptor signaling. In line with this, intraoperative vasoresponsiveness correlated with increased postoperative norepinephrine requirements, supporting clinical relevance of the observed hyporesponsiveness.

Long-standing adrenergic (over)stimulation downregulates myocardial β_1_/β_2_-adrenergic receptors in HF [9,19]. Our findings extend this to vascular α_1_-adrenergic signaling: phenylephrine responses were preoperatively reduced in HF and absent ex vivo, supporting α_1_-receptor impairment [7,8,9,20]. A prior systematic review did not find consistent evidence for vascular α_1_-receptor downregulation in HF, likely reflecting differences in receptor subtype expression and technical limitations [20,21]. Two clinical studies reported mixed results regarding α_1_-agonist responses [16,22]. We hypothesize that this may reflect differences in patient selection and medication use. In our study, vasopressin responses were increased in HF patients ex vivo, further supporting that the dominant alteration is more likely α_1_-receptor specific. Our findings demonstrate attenuated in vivo α_1_-adrenergic vasoresponsiveness in patients with HF, with ex vivo observations suggesting a similar pattern. Of note, despite the abolished ex vivo vasoresponsiveness, HF patients still exhibited some vascular reactivity in vivo. Certainly, the absence of measurable contraction in isolated vessels does not necessarily indicate an absent systemic response to phenylephrine. The 26% increase in SVR in patients with HF, compared with 51% in controls, demonstrates attenuated rather than abolished in vivo responsiveness. Variability in receptor expression in the various vascular beds may provide a plausible explanation for this discrepancy, although our study cannot establish this [20].

Phenylephrine responsiveness transiently increased after induction in both groups, likely reflecting anesthesia-related effects, but overall remained depressed in HF. On postoperative day 1, phenylephrine responsiveness declined significantly in both cohorts. Beyond perioperative inflammation and CPB-related vasoplegic mechanisms [12,23,24], this postoperative decline is consistent with an intraoperative catecholamine surge in response to cardiac surgery that may lead to temporary impairment of α_1_-adrenergic receptors even in controls [25]. In HF patients, the reduced preoperative α_1_-reserve may render vascular tone particularly vulnerable after CPB, explaining the near-abolition of phenylephrine responsiveness [26].

HF arterioles exhibited increased ex vivo sensitivity to vasopressin. Chronic HF is associated with altered vasopressin signaling and elevated copeptin levels [27]. High preoperative copeptin levels reflect activation of the vasopressin system, and sustained activation may lead to depletion of the pituitary stores, explaining why it identifies patients who benefit from perioperative vasopressin administration [11,28]. Our ex vivo findings align with possible relative endogenous vasopressin depletion in HF. Although our sample size is small and our results should be interpreted with caution, the greater ex vivo responsiveness to vasopressin in this group may provide a physiological rationale for supporting vasopressin as a targeted therapy when catecholamines fail or even as a first-line agent. This finding complements clinical evidence from Cui et al. and the VANCS trial supporting early vasopressin-based treatment in postoperative vasoplegia [29,30]. However, a previous retrospective study in patients with preoperative LV dysfunction has shown less favorable results; therefore, a clinical advantage with this therapy should not be assumed [31]. Thus, the clinical benefit of this approach requires prospective evaluation in this specific patient population.

Inducible nitric oxide (NO) synthase-derived NO possibly contributes to vasoplegia [5,32]. Methylene blue, a cGMP-pathway inhibitor, is used as rescue therapy, although evidence remains limited [33]. No group differences in nitroglycerin responsiveness were observed, indicating that altered NO-mediated vasodilation is unlikely to explain the higher vasoplegia risk in HF [34,35].

The ΔSVR after CPB was associated with postoperative norepinephrine requirements, indicating that intraoperative hyporesponsiveness may identify patients at risk for persistent hypotension and hemodynamic instability. Real-time vasoresponsiveness assessment could help guide therapy, counteracting the incidence of systemic hypotension [36]. Moreover, our findings of diminished α_1_-adrenergic responsiveness to phenylephrine suggest that in HF patients, perioperative reliance on α_1_-agonists to maintain hemodynamic stability may be less effective. These observations raise the hypothesis that earlier use of non-adrenergic vasopressors, such as vasopressin, could be beneficial; however, this requires confirmation in adequately powered randomized controlled trials.

## 5. Limitations

Limitations include missing in vivo data (9% phenylephrine, 14% nitroglycerin) due to prevailing clinical safety, possibly underestimating between-group differences. The surgical population was heterogeneous by design in order to reflect real-world cardiac surgery practice with relevant HF as the common denominator. As a result, medication- and procedure-related confounding cannot be fully excluded. Adjustment for procedure type was not performed given the limited cohort size and the variety of procedures performed. However, diminished α_1_-adrenergic responsiveness was already present before surgery, and a similar pattern was observed during ex vivo vascular testing. These findings suggest that this phenotype is pre-existent and is related to the pathophysiology of chronic HF. Procedural factors may have contributed to the further attenuation in vascular responsiveness postoperatively, although their effects cannot be determined in the current study. Nevertheless, CPB and aortic cross-clamp times were similar between the two groups, which represent major determinants of surgical complexity and surgical trauma. The effects of preoperative therapy also cannot be excluded. It is important to highlight that the ex vivo sample size was small, and even though group differences were clear and in line with in vivo observations, these findings should be confirmed in larger cohorts.

In addition, PulseCO calibration was not possible before induction, possibly affecting baseline SVR accuracy, although relative SVR changes were consistent. Furthermore, patients undergoing LVAD implantation contributed to the postoperative measurements (*N* = 4 at T3, *N* = 3 at T4). Pulse-contour-derived cardiac output in this setting might introduce potential measurement error into the calculated SVR responses. Importantly, vasoreactivity was assessed as the relative change in SVR from each patient’s own pre-intervention value. Between-group comparisons therefore assessed differences in within-patient responses to phenylephrine rather than differences in absolute SVR values.

Overestimation of LV function in controls undergoing mitral valve repair due to severe mitral regurgitation may be possible; however, pre/postoperative comparisons did not show significant LV function decline [37]. HF patients more frequently received milrinone and dobutamine perioperatively, drugs known to induce peripheral vasodilation. Although attenuated phenylephrine responsiveness was already evident before surgery and a similar pattern was observed ex vivo, the net effect on vasodilation of milrinone and dobutamine cannot be quantified. Moreover, given the small study size, multivariable adjustment was limited and thus, residual confounding affecting the association between intraoperative vasoresponsiveness and postoperative norepinephrine requirements cannot be fully excluded. Similarly, their effect on vasodilation during the first postoperative day cannot be excluded. Additionally, although we expect that the postoperative measurements occurred within a similar time frame across patients, the exact timing was not recorded. We therefore cannot confirm comparable timing between groups, and even though we do not expect that small variations in the timing would have influenced our findings, we cannot exclude a potential influence. The same consideration applies to the ex vivo experiments. Although the experiments were performed the morning after the procedure, the exact storage duration of the tissue samples was not recorded and thus, its influence on the experiments performed cannot be entirely excluded. Considering our small sample size and its inherent restrictions, our results should be confirmed in larger cohorts. Furthermore, based on this study, we cannot distinguish whether the diminished vasoresponsiveness is the result of receptor desensitization or downregulation, and this should be further investigated in future studies. Lastly, for the definition of postoperative vasoplegia, we used a CI ≥ 2.2 L/min/m^2^ and may therefore have failed to identify patients with a combined hemodynamic profile. This could lead to underestimation of vasoplegia incidence. This limitation should be considered when interpreting postoperative vasoplegia incidence [2,38].

## 6. Conclusions

HF patients exhibited diminished vasoresponsiveness to phenylephrine consistent with α_1_-dependent impairment. This reduced α_1_-mediated vasoresponsiveness was present at baseline and was nearly abolished post-CPB. Intraoperative vasoresponsiveness correlated with postoperative norepinephrine requirements, suggesting an association with hemodynamic instability. Conversely, HF arterioles exhibited heightened ex vivo sensitivity to vasopressin. These results provide a physiological rationale suggesting potential benefit from vasopressin-based therapy in postoperative systemic hypotension. Given the exploratory nature of this study and the small cohort size, these observations require confirmation in prospective randomized studies involving clearly selected patient populations. These findings advance understanding of vasoplegia pathophysiology in HF and support the evaluation of HF-tailored targeted preventive and therapeutic strategies.

## Figures and Tables

**Table 1:** Baseline characteristics.

	Heart Failure (*n* = 18)	Control (*n* = 18)
Age (years)	68 (62–71)	64 (59–69)
Male sex (%)	12 (67)	16 (89)
Body mass index (kg/m^2^)	27 ± 4	24 ± 3
Diabetes (%)	7 (39)	1 (6)
Prior hypertension (%)	7 (39)	3 (17)
Pulmonary hypertension (%)	5 (28)	2 (11)
Previous cardiac surgery (%)	4 (22)	1 (6)
Hemoglobin (mmol/L)	8.5 ± 1.0	9.1 ± 1.0
Mean arterial pressure (mmHg)	83.1 ± 14.7	95.3 ± 17.2
Cardiac output (L/min)	5.7 ± 1.5	5.9 ± 1.1
Systemic vascular resistance (dynes⋅sec⋅cm^−5^)	1147.9 ± 327.9	1243.7 ± 309.9
Creatinine clearance (mL/min/1.73 m^2^)	67 ± 20	78 ± 16
EuroSCORE II (%)	9.8 (6.6–15.5)	1.5 (1.1–2.7)
Left ventricular ejection fraction (%)	24.9 ± 6.4	60.3 ± 6.9
C-reactive protein (mg/L)		
<3 (%)	2 (11)	1 (6)
3–10 (%)	12 (67)	16 (89)
10–100 (%)	4 (22)	1 (6)
>100 (%)	0	0
White blood cell count (×10^9^/L)	9 ± 3.4	6.7 ± 1.7
Bilirubin total (μmol/L)	12 ± 5.9	12.8 ± 7.9
Medication use		
Betablocker (%)	16 (89)	6 (33)
Angiotensin-converting enzyme inhibitor/angiotensin receptor blocker (%)	11 (61)	9 (50)
Mineralocorticoid receptor antagonist (%)	8 (44)	2 (11)
Diuretic (%)	16 (89)	5 (28)
Inotropes (milrinone/dobutamine) (%)	2 (11)	0

Data are presented as mean ± SD, median (IQR), or number (%).

**Table 2:** Intraoperative characteristics.

	Heart Failure (*n* = 18)	Control (*n* = 18)
Mitral valve plasty (%)	10 (56)	17 (94)
Tricuspid valve plasty (%)	6 (33)	9 (50)
Surgical left ventricular restoration (%)	7 (39)	0
Left ventricular assist device implantation (%)	4 (22)	0
Coronary artery bypass grafting (%)	7 (39)	1 (6)
Aortic valve replacement (%)	3 (17)	0
Aorta surgery (%)	2 (11)	1 (6)
Hypothermia during CPB (°C)	32 (32–37)	32 (30–33)
CPB time (min)	174 (120–214)	143 (103–225)
Cross clamp time (min)	135 ± 37	132 ± 52
Procedure time (min)	300 (254–388)	243 (188–326)

Data are presented as mean ± SD, median (IQR), or number (%). CPB: cardiopulmonary bypass.

**Table 3:** Postoperative characteristics.

	Heart Failure (*n* = 18)	Control (*n* = 18)	*p* Value
C-reactive protein (mg/L)	66.2 (47.3–84.9)	63.1 (40.4–70.3)	0.343
White blood cell count (×10^9^/L)	15.1 ± 4.6	12.4 ± 4.1	0.072
Lactate (mmol/L)	2.2 (1.8–3.5)	1.9 (1.3–2.3)	0.059
MAP 0–24 h	72 ± 8	77 ± 5	0.037
CI 0–24 h	2.3 ± 0.4	2.5 ± 0.6	0.217
Vasoplegia (%)	0	1 (6)	1.000
ICU time (days)	2 (1–3)	1 (1–2)	0.133
Medication use			
Epinephrine	5 (28)	0	0.045
Norepinephrine	17 (94)	10 (56)	0.018
Dobutamine	17 (94)	5 (28)	<0.001
Milrinone	17 (94)	3 (17)	<0.001
Terlipressin	3 (17)	0	0.021
Max NE dosage on ICU (μg/kg/min)	0.18 (0.08–0.55)	0.03 (0–0.13)	0.002
Total duration NE on ICU (min)	1281 (215–1615)	177 (0–882)	0.009
Total NE dosage on ICU (μg/kg)	187 (14.8–345)	3.61 (0–41.6)	0.003
NE on ICU 0–24 h (μg/kg)	170 (14.8–319)	3.61 (0–41.6)	0.003

Data are presented as mean ± SD, median (IQR), or number (%). CI: cardiac index, ICU: intensive care unit, MAP: mean arterial pressure, NE: norepinephrine.

**Table 4:** Association between %ΔSVR at baseline (T1), after induction (T2) and after cardiopulmonary bypass cessation (T3) with the total amount of norepinephrine given during the first 24 h. Univariate and multivariate regression models, adjusted for heart failure and norepinephrine dosage at that time point.

	Beta Coefficient	95% CI	R^2^
Univariable			
ΔSVR at T1	−259.4	−554.5–35.8	0.094
ΔSVR at T2	−65.5	−264.4–133.3	0.014
ΔSVR at T3	−346.6	−541.2–−151.9	0.291
Multivariable			
ΔSVR at T1	−75.4	−398.6–247.7	0.252
ΔSVR at T2	33.4	−166.6–233.4	0.203
ΔSVR at T3	−270.2	−519.3–−21.1	0.361

CI: confidence interval, ΔSVR: systemic vascular resistance change.

## Data Availability

The data presented in this study can be made available upon reasonable request from the corresponding author due to data privacy rules.

## Supplementary Materials

The following supporting information can be downloaded at: https://www.mdpi.com/article/10.3390/jcm15208179/s1, Anesthetics and hemodynamics; Ex vivo; Figure S1: Flowchart study; Figure S2: Patient selection flow diagram (in-vivo); Figure S3: Patient and biopsy selection flow diagram (ex-vivo); Figure S4: Vasoconstriction test; Figure S5: Vasodilation test; Table S1: Baseline characteristics of the ex-vivo population; Table S2: Absolute delta systemic vascular resistance (ΔSVR) with 95%CI. A sensitivity analysis.

## Abbreviations

CO	Cardiac output
CPB	Cardiopulmonary bypass
HF	Heart failure
LVEF	Left ventricular ejection fraction
MAP	Mean arterial pressure
NO	Nitric oxide
SVR	Systemic vascular resistance
ΔSVR	Systemic vascular resistance change
