# Supplementary material for: Impact of Chronic Heart Failure on Vascular Responsiveness: A Prospective Observational In Vivo and Ex Vivo Study in Patients Undergoing Cardiac Surgery
Source: J Clin Med. 2026 Oct 21;15(20):8179. doi: 10.3390/jcm15208179 (PMC13706177; doi:10.3390/jcm15208179)
Supplement: Supplementary file 1 [file jcm-15-08179-s001.zip › Supplement_clena.pdf]

Supplement

Anesthetics and hemodynamics

All patients received anesthesia according to a standardized protocol, using target-controlled infusion of propofol and remifentanil or sufentanil. Cannulation of the radial artery was performed in all patients before induction for invasive hemodynamic blood pressure monitoring. A central venous catheter was inserted in the internal jugular vein and a flow-directed balloon-tipped pulmonary artery catheter was introduced after induction. PulseCO™ software (LiDCO, London, UK) was used for measurement of cardiac output and systemic vascular resistance. Cardiac output measured using a pulmonary artery catheter connected to a Vigilance II monitor (Edwards Lifesciences, Irvine, CA, USA) served as the reference for calibrating the PulseCO-derived cardiac output measurements obtained with the LiDCOplus system (LiDCO Ltd, London, UK). Vasoreactivity tests were analyzed offline using LiDCOviewSE software (LiDCO Ltd, London, UK).

Intra- and postoperatively, norepinephrine 0.04–0.2 μg/kg/min was started if the mean arterial pressure was ≤ 65 mmHg and the cardiac index was normal (after adequate administration of intravascular fluids if necessary), aiming for a mean arterial pressure > 65 mmHg and adequate end-organ perfusion. When a norepinephrine dosage >0,5 μg/kg/min was required, terlipressin was started. The vasoactive medication was reduced when the mean arterial pressure was > 65 mmHg in combination with adequate end-organ perfusion. In this situation, terlipressin was reduced first.

Patients did not receive angiotensin-converting-enzyme (ACE) inhibitors, angiotensin receptor blockers and diuretics on the day of surgery.

Ex-vivo

Resistance arteries (~300 μm) were dissected, isolated, and stored overnight in cold, oxygenated (95% O2–5% CO2) Krebs bicarbonate solution (in mM: 118 NaCl, 4.7 KCl, 2.5 CaCl2, 1.2 MgSO4, 1.2 KH2PO4, 25 NaHCO3, 8.3 glucose; pH 7.4). The next day, segments (~2 mm) were mounted in microvascular myographs (Danish MyoTechnology, Aarhus, Denmark) with 6-ml organ baths containing Krebs bicarbonate solution (95% O2–5% CO2) at 37°C. Contractile force was recorded with a Harvard isometric transducer following a 30-minute equilibration.

After normalization, maximum contractile responses were determined using 100 mM KCl [1]. Maximum KCl responses in phenylephrine experiments were 14.8 ± 9.1 mN (control) and 17.3 ± 8.2 mN (HF). In vasopressin experiments, maximum KCl responses were 10.9 ± 6.2 mN (control) and 16 ± 13.4 mN (HF). KCl was then washed out. Concentration-response curves were constructed to phenylephrine (30 pM - 10 nM, equivalent to 3×10^{-8} - 1×10^{-5} mmol/L) and vasopressin (0.1 pM - 30 pM, equivalent to 1×10^{-10} - 3×10^{-8} mmol/L) and expressed as a percentage of 100 mM KCl-induced contractions.

Supplemental Figures

Figure S1 Flowchart study

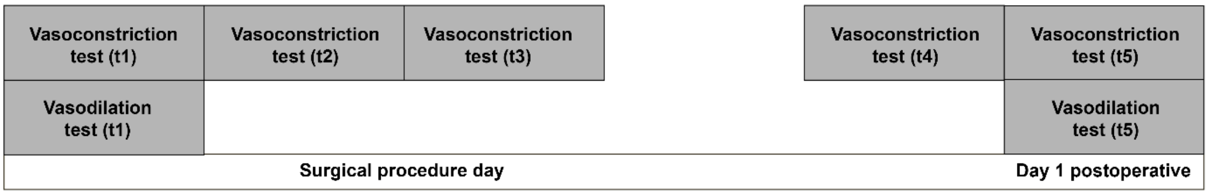

Figure S2 Patient selection flow diagram (In-vivo)

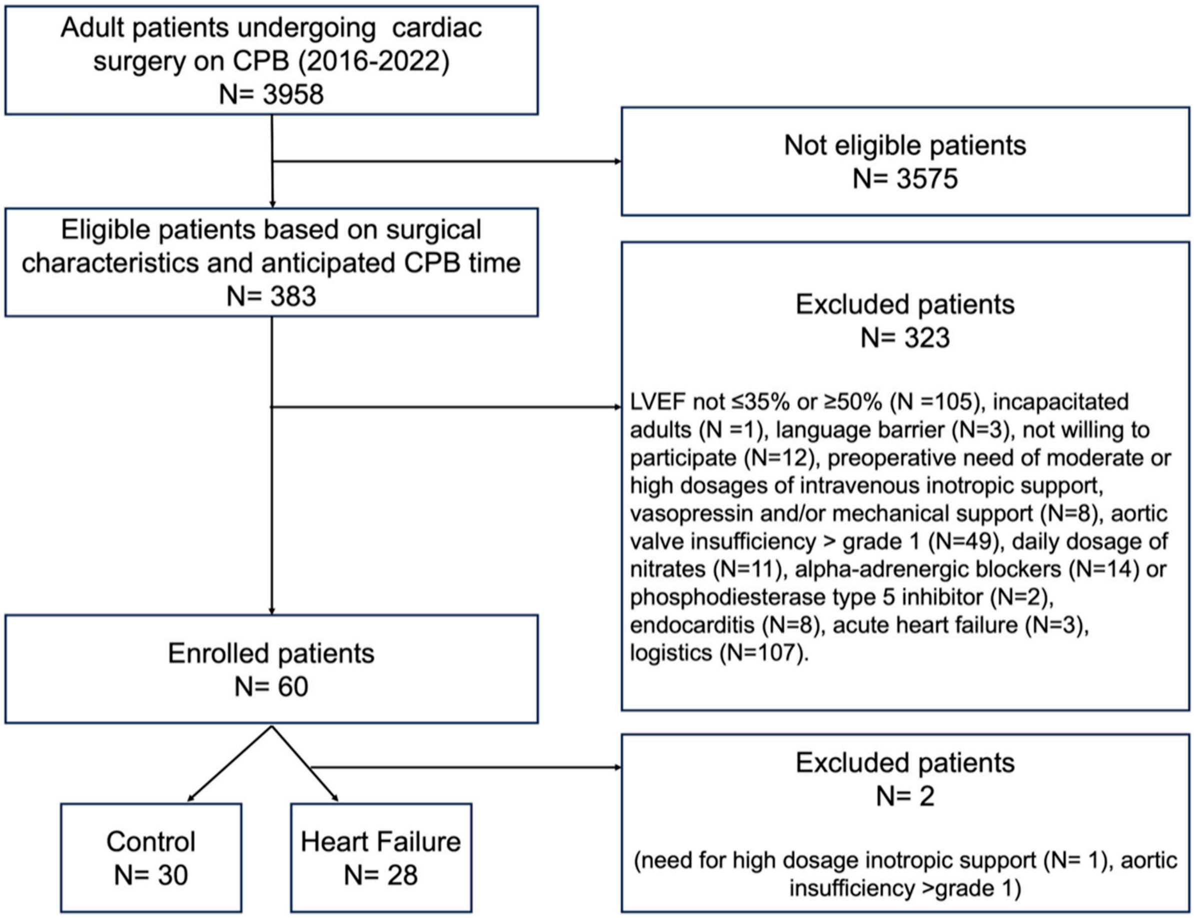

Figure S3 Patient and biopsy selection flow diagram (ex-vivo)

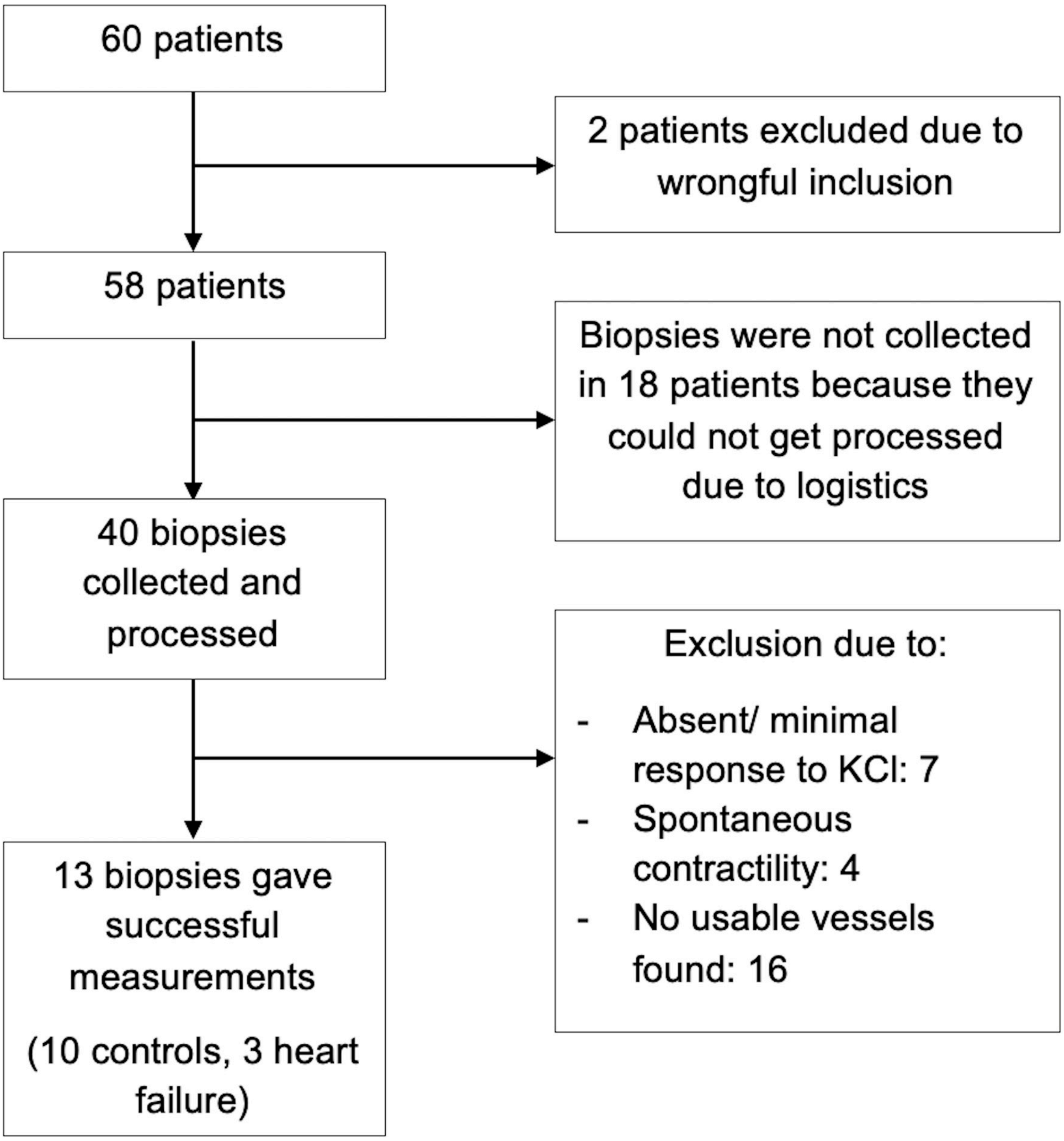

Figure S4 Vasoconstriction test

A. Change in cardiac index

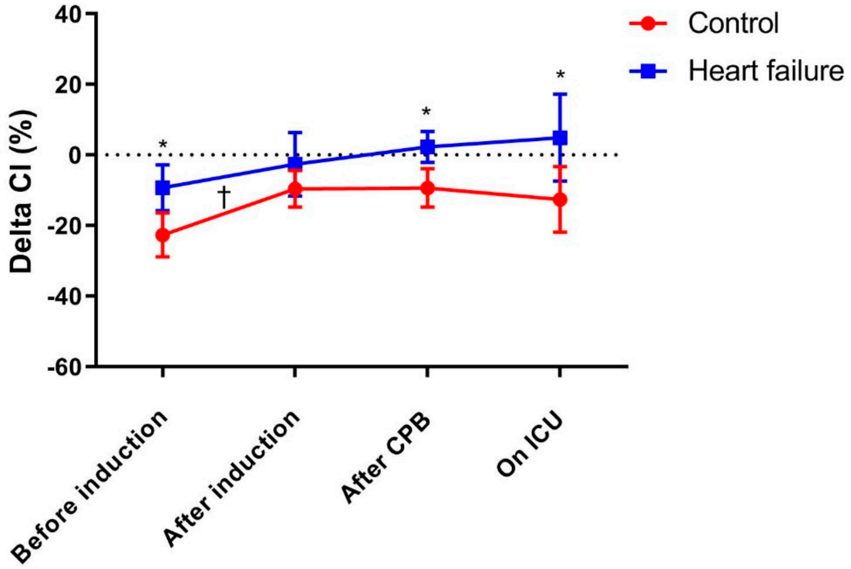

Patients					
Control	—	16	17	17	14
Heart failure	—	17	17	17	16

B. Change in mean arterial pressure

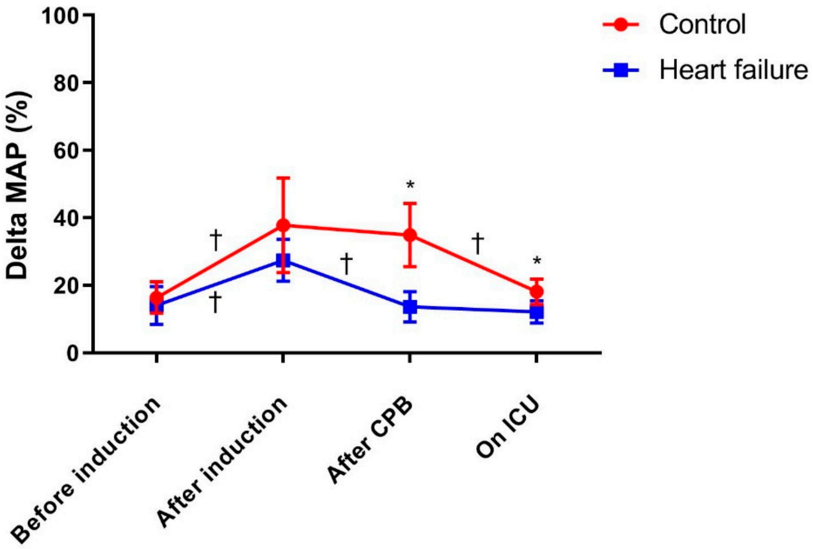

Patients					
Control	—	16	17	17	14
Heart failure	—	17	17	17	16

Delta systemic cardiac index (CI, %) and mean arterial pressure (MAP, %) with 95%CI. * Significant difference ($P<0.05$) between groups; † significant difference between timepoints.

Figure S5 Vasodilation test

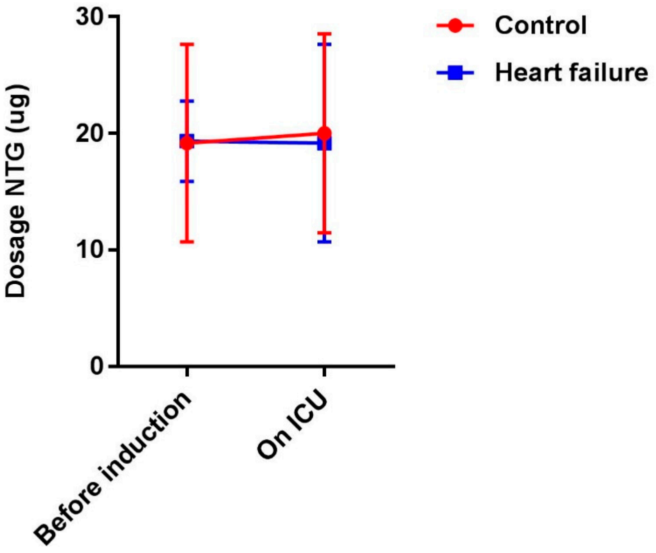

Patients		Before induction	On ICU
Control	—	12	13
Heart failure	—	15	12

Dosage nitroglycerine (with 95% CI) needed for 10% decrease in MAP.

Supplementary Tables

Table S1 Baseline characteristics of the ex-vivo population

	**Heart failure (n=3)**	**Control (n=10)**
Age (years)	66 (56-72)	67 (63-76)
Male sex (%)	100	40
Body mass index (kg/m^2)	29 (28-33)	25 (23-27)
Diabetes (%)	67	0
Prior hypertension (%)	67	40
Pulmonary hypertension (%)	67	40
Previous cardiac surgery (%)	0	0
Hemoglobin (mmol/l)	9.3 (8.4-9.4)	9 (8.2-9.6)
Systolic blood pressure (mmHg)	119 (110-165)	148 (138.3-160.8)
Diastolic blood pressure (mmHg)	71 (66-71)	76 (71.8-87.8)
Mean arterial pressure (mmHg)	87 (81-102)	100.5 (93.5-108.8)
Creatinine clearance (ml/min/1,73m^2)	77 (39-78)	78 (68.8-84.8)
EuroSCORE II (%)	9.8 (7.8-9.5)	1.3 (0.8-3)
Left ventricular ejection fraction (%)	21 (19-22)	61 (59-66)
C-reactive protein (mg/L)		
≤3 (%)	33	100
3-10 (%)	33	0
10-100 (%)	33	0
>100 (%)	0	0
White blood cell count ($^*10^9$/L)	6.9 (4.3-9.2)	6.5 (5.9-8.1)
Bilirubin total (µmol/L)	10 (7-21)	13 (9-18)
Betablocker (%)	100	30
Angiotensin-converting enzyme inhibitor/ angiotensin receptor blocker (%)	67	30
Mineralocorticoid receptor antagonist (%)	33	10
Diuretic (%)	100	30
Inotropes (milrinone/ dobutamine) (%)	0	0

Data are presented as median and range or median and IQR for heart failure and control respectively.

Table S2. Absolute delta systemic vascular resistance (ΔSVR) with 95%CI. A sensitivity analysis

**Time point**	**Controls, mean (95% CI)**	**HF, mean (95% CI)**	**HF − control (95% CI)**	**P value**
Baseline	635.8 (457.1 - 814.5)	310.7 (166.5 - 454.9)	-325.1 (-554.7 - -95.5)	0.006
After anesthesia induction	751.1 (518.4 - 983.8)	575.5 (368.2 - 782.8)	-175.6 (-487.3 - 136.1)	0.270
After cessation of CPB	689.9 (462.5 - 917.4)	136.5 (−1.9 - 275.0)	-553.4 (-819.7 - -287.1)	<0.001
First postoperative day	308.0 (225.5 - 390.5)	114.6 (42.0 - 187.1)	-193.4 (-303.3 - -83.6)	<0.001

Mean absolute ΔSVR in dyn·s·cm^{-5}

CPB: cardiopulmonary bypass, HF: heart failure

References

1. Bridges LE, Williams CL, Pointer MA, Awumey EM. Mesenteric artery contraction and relaxation studies using automated wire myography. J Vis Exp. 2011;3119.
